# Toward the Identification of Neurophysiological Biomarkers for Alzheimer’s Disease in Down Syndrome: A Potential Role for Cross-Frequency Phase-Amplitude Coupling Analysis

**DOI:** 10.14336/AD.2022.0906

**Published:** 2023-04-01

**Authors:** Daniella B Victorino, Jean Faber, Daniel J. L. L Pinheiro, Fulvio A Scorza, Antônio C. G Almeida, Alberto C. S Costa, Carla A Scorza

**Affiliations:** ^1^Discipline of Neuroscience, Department of Neurology and Neurosurgery, Federal University of São Paulo / Paulista Medical School, São Paulo, SP, Brazil.; ^2^Department of Biosystems Engineering, Federal University of São João Del Rei, Minas Gerais, MG, Brazil.; ^3^Division of Psychiatry, Case Western Reserve University, Cleveland, OH, United States.; ^4^Department of Macromolecular Science and Engineering, Case Western Reserve University, Cleveland, OH, United States.

**Keywords:** Down syndrome, Alzheimer’s disease;, brain oscillations, phase-amplitude coupling, EEG biomarkers

## Abstract

Cross-frequency coupling (CFC) mechanisms play a central role in brain activity. Pathophysiological mechanisms leading to many brain disorders, such as Alzheimer’s disease (AD), may produce unique patterns of brain activity detectable by electroencephalography (EEG). Identifying biomarkers for AD diagnosis is also an ambition among research teams working in Down syndrome (DS), given the increased susceptibility of people with DS to develop early-onset AD (DS-AD). Here, we review accumulating evidence that altered theta-gamma phase-amplitude coupling (PAC) may be one of the earliest EEG signatures of AD, and therefore may serve as an adjuvant tool for detecting cognitive decline in DS-AD. We suggest that this field of research could potentially provide clues to the biophysical mechanisms underlying cognitive dysfunction in DS-AD and generate opportunities for identifying EEG-based biomarkers with diagnostic and prognostic utility in DS-AD.

Brain function and cognition rely on the precise coordination of neural activity within and across brain areas. Yet, how neural networks achieve this precise coordination is part of a long-standing debate in neuroscience, and much remains to be understood about how brain function emerges from different patterns of neuronal communication in both health and disease.

Early research efforts were often devoted to investigating the functional properties of frequency bands in isolation; however, many recent studies have focused on exploring how distinct brain rhythms could interact with each other to support information processing and communication within the brain [1-3]. Emerging evidence has shown that the dynamic modulation between brain subsystems that oscillate at different frequency bands can be assessed by estimating cross-frequency coupling (CFC), which refers to the interaction between oscillations at distinct frequencies. Such inter-frequency interaction can be achieved, for example, via phase-amplitude coupling (PAC), wherein the amplitude (or power) of a fast oscillation is modulated by the phase of a slower oscillation [4, 5]. The modulation of gamma amplitude by theta phase, which is the most-studied type of PAC, has been implicated in multiple cognitive processes [5-10], and alterations in this interaction have been reported in many brain disorders [11-13]. For example, it has been suggested that cross-frequency interactions between neural oscillations may be impaired in early stages of Alzheimer’s disease (AD) [14-17], and such changes have also been associated with the progression from mild cognitive impairment (MCI) to AD and dementia [18, 19].

Individuals with Down syndrome (DS) are at a greater risk of developing early-onset dementia, which is driven by the near-universal presence of AD-type pathology in DS brains by 40 years of age [20, 21]. Establishing the natural sequence of cognitive decline due to AD has proven challenging in the population of individuals with DS, especially because the extent of premorbid intellectual disability is frequently unknown and highly variable among these individuals [22, 23]. Although cerebrospinal fluid (CSF), plasma, and neuroimaging biomarkers have proven useful in the diagnosis of early-onset AD in DS (DS-AD) [24], the potential role of non-invasive electroencephalography (EEG)-based biomarkers for detecting DS-AD is still underexplored.

Here, we briefly review some of the basic physiological mechanisms underlying neural oscillations, and how dynamic interactions between different brain rhythms can be studied by analysis of CFC in both health and disease. We then review preclinical and clinical studies aimed at investigating electrophysiological alterations that may be associated with AD progression. Particular focus was given to studies on the potential use of theta-gamma PAC as a biomarker of cognitive decline in AD. We argue that PAC analysis has great potential as an adjuvant tool for evaluating clinical changes in brain network dynamics associated with early stages of AD in the DS population, as well as in preclinical and clinical research aimed at exploring electrophysiological mechanisms associated with the progressive neurodegenerative processes in DS-AD. We conclude by speculating that this line of investigation could also provide an opportunity for the identification of non-invasive, EEG-based biomarkers with diagnostic and prognostic utility in DS-AD.

## Network dynamics underlying brain oscillations

Brain oscillations result from rhythmic fluctuations in the electrophysiological activity of single neurons, local assemblies of neurons, and/or several spatially distributed neuronal assemblies [25]. The primary mechanism underlying the generation of oscillatory activity is movements of ionic species across cell membranes. These ionic flux dynamics produce voltage fluctuations in membrane potential (Vm) between two main states: the hyperpolarized (‘down’) state, wherein membrane potentials are typically far from action potential threshold, and the depolarized (‘up’) state, wherein the arrival of postsynaptic potentials interrupts periods of quiescence [26]. The superposition of ionic currents from all active membranes gives rise to field potentials, which can be extracellularly recorded by many measurement modalities, such as local field potentials (LFP), electro-corticography (ECoG), scalp EEG, and magnetoencephalography (MEG) [27]. Field potentials are measured noninvasively by EEG and MEG recordings, whereby electrodes and sensors are placed at the scalp and outside the head, respectively. On the other hand, ECoG and LFP signals are recorded invasively, and while ECoG involves placing electrodes either outside (epidural) or under (subdural) the dura mater, LFP involves inserting electrodes into the brain [27].

Synchronous activity of neural networks generates rhythmic voltage oscillations at multiple frequencies, which are conventionally classified as ultraslow (<1 Hz), delta (1-4 Hz), theta (4-8 Hz), alpha (8-12 Hz), beta (12-30 Hz), slow gamma (30-80 Hz), fast gamma (80-200 Hz), and ultra-fast gamma (200-600 Hz) oscillations [25]. Switches between brain states are underlined by changes in the global pattern of neural activity, and these state-dependent changes can be well distinguished by electrophysiological recordings [28]. For example, during wakefulness, EEG typically shows low-voltage fast activity, and, as an individual falls asleep, the EEG progressively changes into a slow, high-amplitude wave pattern [28]. Dynamic changes in EEG pattern can also occur in a task-dependent manner. For example, while theta oscillations have been associated with spatial navigation and memory [29, 30], oscillatory activity at gamma frequencies has been found to underlie selective attention and information transmission [31, 32].

Timing in neural networks is mainly determined by the activity of gamma-aminobutyric acid (GABA) inhibitory interneurons [33]. The time-dependent manner by which inhibitory activity silences principal neurons allows for a ‘window of opportunity’, in which excitatory inputs can sum and reach the threshold for an action potential generation. In fact, cyclical variations in neuronal excitability not only affect the likelihood of spike output, but also the sensitivity to synaptic inputs [34]. Synaptic inputs consistently arriving at the phase of maximum excitability may benefit from effective connectivity [34, 35]. The mechanism of “communication through coherence” suggests that neuronal communication is optimized when activated neuronal groups communicate in temporal windows of coherence, i.e., information transmission between neuronal groups is facilitated if there is phase synchronization between their oscillatory activity [34, 35].

Coherence may reflect coupling between spikes in one region and the LFP within either the same or a different region (i.e., spike-field coherence), as well as phase relationship between neural oscillations at two different brain regions [34, 35]. For example, hippocampal gamma-band coherence was found to be increased during the encoding of subsequently well recognized stimuli in adult rhesus monkeys [36]. Similarly, gamma-band coherence between hippocampal CA3 and CA1 regions was found in rats during a delayed spatial alternation task, wherein peak increases in coherence were observed when rats approach the maze segment associated with memory retrieval [31]. In addition, compelling evidence has shown that changes in synchronization of oscillatory activity between neural groups can support flexible communication between them (i.e., selective communication through selective coherence), wherein synaptic inputs compete with each other for target entrainment [35]. These findings suggest dynamic changes in coherence as an important mechanism by which neural networks adapt to both the internal and the external environments, as well as to cognitive demands [34, 35].

While “communication through coherence” contemplates coupling between oscillatory activity at the same frequency, more complex patterns of network organization can arise from mechanisms by which oscillators across different frequency bands can be nested within each other [4, 5].

## The potential role of CFC as a fundamental mechanism to coordinate neural activity and processing of information across the brain

Because brain rhythms coexist in different frequency bands, their physiological role in cognitive functions cannot be fully appreciated in isolation. Therefore, it is critical to understand the production of CFC, i.e., how different brain rhythms interact with each other. Neural oscillations can be hierarchically organized in such a way that the phase or amplitude of a slower oscillation modulates the phase or amplitude of a faster one via phase-phase, amplitude-amplitude, or phase-amplitude coupling [7, 8, 37]. These properties allow oscillators at different frequencies to interact with each other both locally and across brain networks [4, 5, 29, 38].

CFC has been associated with multiple brain and behavioral states. For example, in awake rats, it was found that the amplitude of high frequency oscillations was modulated by the phase of ongoing theta rhythm, both within and across striatal-hippocampal circuits, and that the strength of such CFCs dynamically changed according to the rodent’s behavioral performance [37]. In addition, CFC has also been used to infer direction of communication between brain structures, wherein inter-regional CFC is calculated from a pair of brain networks forming a driver-receiver relationship [39, 40]. More recently, a functional link has been suggested between CFC and “communication through coherence” as it was found that long-range synchronization at faster frequencies was modulated as a function of the phase of slower oscillations, which support a role for CFC in long-range information transfer by facilitating inter-regional synchronization of faster rhythms [41].

**Figure 1.**
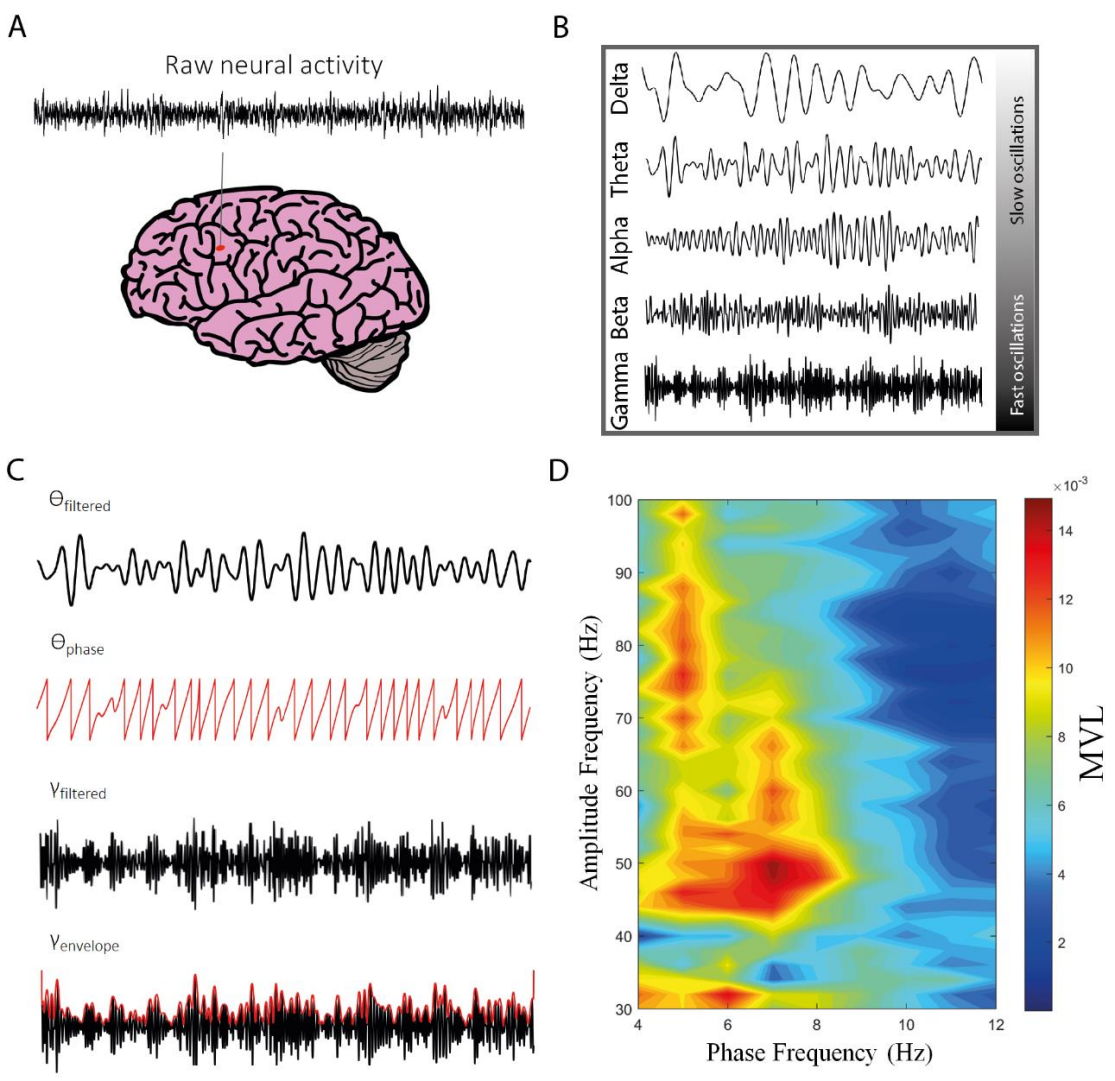
Schematic illustration of the workflow for assessing phase-amplitude coupling (PAC). (A) The first step consists of recording the electrical activity of the brain. Raw neural activity can be recorded by several measurement modalities, such as local field potentials (LFP), electrocorticography (ECoG), scalp EEG, and magnetoencephalography (MEG). (B) Raw neural signals are then subjected to preprocessing techniques, wherein unwanted artifacts (e.g., motion and power line artifacts) are removed and oscillations at frequency ranges of interest are extracted (e.g., delta (δ, 1-4 Hz), theta (ϴ, 4-8 Hz), alpha (α, 8-12 Hz), beta (β, 12-30 Hz), and gamma (γ, 30-80 Hz) oscillations). (C) By using the Hilbert transform, for example, the instantaneous phase of the slow oscillation (e.g., theta), as well as the amplitude (envelope) of the faster oscillation (e.g., gamma), are estimated. Both estimations are then used to construct the empirical phase-amplitude distribution, which provides the amplitude of the fast oscillation at each phase of the slow oscillation. Many approaches have been described for PAC estimation, such as the modulation index (MI), the phase-locking value (PLV), and the mean vector length (MVL) modulation index. (D) The level of coupling between several frequency pairs can be expressed by the phase-amplitude comodulogram plot, wherein the phase frequency is represented in the x-axis and the amplitude frequency is represented in the y-axis. The pseudocolor scale in the right represents PAC intensity as measured by MVL.

As aforementioned, the coupling between theta phase and gamma amplitude (theta-gamma PAC) is the best-studied modality of CFC [5, 7]. It describes the statistical dependence of the amplitude (or power) of gamma oscillations on the phase of theta rhythm, and it has been proposed as an effective mechanism to coordinate neural activity across different spatial and temporal scales [5, 7, 8, 42-44] (see Fig. 1 for further details on PAC analysis workflow). Theta-gamma PAC has been found to be associated with memory reprocessing and consolidation during REM sleep [45], as well as with working memory [2, 3, 8, 46-48] and long-term memory processes [6, 49].

The initial idea of a phase-dependent mechanism supporting memory performance stemmed from studies showing theta phase-dependent modulation of synaptic strength and efficacy by using hippocampal long-term potentiation (LTP) and depression (LTD) recordings [50, 51]. This notion has been supported by hundreds of *in vitro* studies showing that theta-burst stimulation (TBS), which consists of multiple pulses at 100 Hz repeated in 5 to 7 Hz inter-burst-intervals (i.e., gamma frequency stimulation bursts repeated at frequencies in the theta range), effectively induces LTP [52, 53]. In addition, in *vivo* studies have shown that phase-locked high-frequency bursts of transcranial magnetic stimulation (TMS) caused an LTP-like increase in motorcortical excitability [54, 55].

The strength of phase-to-power interaction between theta and gamma oscillations may change according to task demand, and it may also predict behavioral performance [56]. For example, in a study by Tort and collaborators [6], these authors showed that the amplitude of low-gamma band became more strongly modulated by the theta phase as the rat’s performance in a learning task improved, and the magnitude of theta-gamma coupling was found to predict correct choice probability. In a different study by Friese et al. [57], scalp-recorded EEG in healthy human subjects provided evidence that PAC between frontal theta and posterior gamma oscillations was enhanced during the encoding of visual stimuli that were subsequently remembered as compared with those that were subsequently forgotten. Task-related changes in theta-gamma PAC have also been observed in subjects performing a working memory task [8, 46-48], wherein the magnitude of this interaction predicted individuals’ working memory performance [47].

Given the potential functional role of theta-gamma PAC in brain activity, special focus has been given to studying the underlying network substrates that give rise to the oscillatory activity at these frequencies [56]. Theta and gamma oscillations are the major rhythms recorded in the hippocampus of freely-behaving rodents [30, 58, 59]. In the CA1 hippocampal network, oscillatory activity in the slow gamma frequency range is driven by CA3 inputs, while gamma activity in a higher frequency range is entrained by inputs from medial entorhinal cortex [58, 60, 61]. There is also strong evidence underscoring the importance of GABA-mediated inhibition in the generation of gamma activity [62, 63].

In the hippocampus, gamma oscillations are generated by two main mechanisms: reciprocal interactions within the highly interconnected GABAergic interneuron network (interneuron network gamma or ING) and feedback loop between excitatory principal neurons and local interneuron networks (pyramidal-interneuron network gamma or PING) [64, 65]. Among multiple GABA-containing interneuron subtypes, fast-spiking, parvalbumin (PV)-expressing basket cells have been considered as an important source of gamma oscillations [62, 63]. Inhibitory synapses between PV basket cells have been found to synchronize electrical activity within this network, whereas PV basket cells establishing perisomatic synapses with principal neurons have been found to distribute the synchronized activity within interneuron network to principal neuron population [66]. In addition, neuronal firing activity of PV basket cells has a high propensity to be phase-locked to theta oscillations in both hippocampus [67] and cortex [68]. Importantly, findings from studies using genetically modified mice suggest that synaptic inhibition onto PV basket cells plays a role in regulating theta-gamma PAC interactions [69]. Although some disagreement exists regarding the genuine functional role of gamma oscillations, this rhythm has been recorded in hippocampal LFP during a variety of behavioral states [58].

Both cholinergic and GABAergic inputs from the medial septal nucleus (MS) contribute to hippocampal theta rhythm generation [49, 70-72]. In fact, temporal control by fast synaptic GABAergic inhibition has been shown to play a role in the regulation of hippocampal theta rhythm, as well as in theta-gamma PAC [30, 62, 64, 73]. Optogenetic silencing of PV basket cells affects spike theta-phase preference of pyramidal cell firing in the hippocampal CA1 area [74]. Infusions of muscimol (a potent GABA_A_ receptor agonist) into MS also affected theta-gamma PAC and reduced the probability of successful memory retrieval [49]. In addition, theta-band oscillatory activity in the hippocampus can also be generated by intrinsic mechanisms as previously showed by studies involving hippocampal slices and isolated rat hippocampus [75, 76]. Recently, Lopez-Madrona and colleagues [77] provided functional evidence supporting the coexistence of independent theta rhythm generators in the hippocampus, each of which can be specifically associated with gamma oscillations, and thus the existence of different theta-gamma frameworks. Importantly, the strength of theta-gamma coupling and the synchronization between theta generators has been shown to increase with novelty and decision-making, which suggests that distinct theta-gamma frameworks may flexibly coordinate information transmission in the hippocampus [77].

The question of whether findings from preclinical electrophysiological studies in animal models can reliably be translated into clinically relevant work is a contentious issue. This is especially true when one considers that brain rhythms are mostly studied by LFP recordings in preclinical research *vis-à-vis* non-invasive, scalp-EEG recordings in clinical setting.

In spite of clear interspecies differences in anatomy and physiology, compelling evidence has shown that the relatively well conserved similarities in brain structure and function between rodents and humans can reasonably support translatability of EEG measures of brain activity across these species. Particularly, the notion that hippocampal theta- and gamma-range responses in humans share many similarities with those in rodents is supported by findings from intracranial EEG recordings in patients with epilepsy surgically implanted with electrodes [78-81]. Hippocampal gamma-frequency activity, as well as task-dependent modulation of gamma activity by the entrained theta cycle, have been observed in both human and rodents [6, 82, 83]. A recent study showed the presence of ~8-Hz oscillations in the posterior area of the human hippocampus, while slower ~3 Hz oscillations were found to be more prevalent in the anterior portion of this structure [78]. Fast theta oscillations were correlated with the speed of movement, while slow theta oscillations did not vary with movement speed, suggesting that hippocampal fast and slow theta oscillations in humans may be functionally analogous to the type 1 and 2 theta oscillations previously observed in rodents [78, 84, 85]. In addition, a prominent theta oscillation was observed during human rapid-eye-movement (REM) sleep [86], and prominent theta oscillations are also seen during REM sleep in rodents [30]. Such oscillations were also found to be phase-coupled to gamma oscillations in human mesiotemporal recordings during REM sleep [87], which closely resembles the phase-amplitude theta-gamma coupling seen in rodents during REM sleep [88, 89].

Beyond the hippocampus, evidence from non-invasive scalp-EEG recordings in human subjects suggests increased cortical theta oscillations in different cortical structures during cognitive tasks (see [90] for a review). This evidence is supported by the hypothesis that hippocampal projections to the neocortex, as well as entrainment of neocortical neurons by hippocampal theta rhythm [68, 91, 92], could drive theta responses in cortical areas, which could then be detectable by non-invasive scalp EEG recordings. Similarly, gamma oscillations have also been recorded (either by scalp electrodes, electrode grids placed subdurally on the cortical surface, or depth electrodes) from different areas of the human cortex and during a variety of behavioral states [93-96].

Although further electrophysiological studies in humans may contribute to the open debate of to what extent theta and gamma oscillations detected by human scalp EEG recordings reflect rhythms generated by neocortical and/or hippocampal areas [90], one cannot ignore the potential that EEG-based measurements have shown as a tool for studying brain rhythms and their dynamic interactions. Accordingly, there has been an increasing interest in the study of theta-gamma PAC as a potential index in neurological and psychiatric disorders [11-13].

## Altered neural activity dynamics in AD

AD is an age-related neurodegenerative disease associated with memory impairment and cognitive decline [97]. The major neuropathologic features of AD are the aggregation of amyloid-β (Aβ) peptides into plaques (and their progressive deposition in brain parenchyma) and the intracellular accumulation of neurofibrillary tangles (NFTs) of tau protein. These processes result in synaptic loss and neuronal death, which eventually translate into the manifestation of AD-related signs and symptoms [97]. AD is also the most common cause of dementia, which is a neurocognitive disorder characterized by decline in memory, language, speech, reasoning, and other cognitive abilities [98]. Dementia ultimately affects people's ability to perform everyday activities, thereby placing a large social and economic burden on communities and health care systems [98].

Given that definitive diagnosis of AD still requires *post-mortem* examination of brain tissue, much of the current research has been directed toward identifying *in vivo* biomarkers that could enable diagnosis of AD during life, and preferentially at its presymptomatic stages, wherein underlying pathophysiological changes are occurring but the disorder is not yet clinically manifested [99-101]. Biomarkers of AD currently used in research and clinical settings include those for Aβ and tau pathology (e.g., CSF and plasma Aβ_42_/Aβ_40_ ratio and phosphorylated-tau, as well as amyloid and tau positron emission tomography (PET)), synaptic loss (e.g., CSF neurogranin and fluorodeoxyglucose (FDG) PET), neurodegeneration (e.g., volumetric magnetic resonance imaging (MRI), and neurofilament light (NfL) concentration in CSF and blood), and inflammation (e.g., CSF YKL-40 and CSF soluble triggering receptor expressed on myeloid cells 2 (sTREM2) and translocator protein (TSPO)) [102, 103]. Although CSF sampling and brain imaging are valuable tools for diagnosing and monitoring AD pathology, these procedures are either invasive, expensive, or rarely available in healthcare facilities.

A particularly promising new approach in the field of AD biomarkers focuses on the monitoring of electrophysiological brain activity and has received growing acceptance as a validated surrogate marker for cognition in the past several years [14, 99, 104]. This concept has been supported by findings showing that the entorhinal cortex and the hippocampus, which are critically involved in spatial and episodic memory, are also particularly vulnerable to AD neuropathology. Therefore, the selective degeneration of synapses in these regions is likely to be an early event in AD pathogenesis [104, 105].

A wide range of brain rhythms has been found to be altered in EEG recordings from subjects with AD. For example, spontaneous EEG recordings from individuals with AD have showed increased power in delta and theta frequency bands and decreases in power of alpha and beta oscillatory activities, which suggest a shift of the power spectrum from faster to slower frequencies [99, 106-109]. Changes in power of gamma oscillations have also been reported in both animal models and persons with AD [110, 111]. Of note, such EEG markers were associated with cortical and hippocampal neurodegeneration as revealed by MRI-based measurements of brain atrophy [17, 112], CSF biomarkers [104, 113], and decline of cognitive performance scores [108, 114]. In addition, alterations in EEG rhythms are also emerging as an objective biomarker for predicting conversion from MCI to AD [107, 115-118].

Recently, EEG markers based on measures of signal complexity (epoch-based entropy) and synchrony (bump modeling) were showed to automatically and efficiently discriminate patients with AD from those with subjective cognitive impairment, MCI, and other pathologies [119]. Yu and collaborators reported that, while the power of EEG harmonic responses to intermittent photic stimulation (PS) was lower in AD subgroups (i.e., patients with mild, moderate, and severe AD), when compared to those with MCI and healthy elderly subjects, the values of multiscale sample entropy were higher. This suggests that the brain's ability to entrain to repetitive stimulation by producing synchronous brain oscillations (and thus reducing EEG signal complexity) is lost in AD patients. The authors also noted that PS-induced changes in EEG complexity varied as a function of disease severity, i.e., from the most prominent changes in brain dynamics in healthy controls to a "transitional change" between MCI and mild AD to an absence of PS-induced changes in mild, moderate, and severe AD [120].

A growing body of evidence suggests that decreased functional connectivity between brain areas may be one of the earliest signatures of AD [121, 122]. Data from AD animal models show impaired cross-frequency modulation of gamma amplitude by the phase of theta oscillations [123-131]. Of note, alterations in theta-gamma PAC were found to arise before Aβ accumulation [127, 128], which suggests that disturbed theta-gamma coupling may be an early event in AD pathology. This hypothesis is supported by clinical evidence showing that, although both subjects with either MCI or AD had decreased levels of theta-gamma PAC compared to healthy controls, individuals with AD had lower levels of this parameter than those with MCI [18]. More recently, a small pilot study using scalp-recorded EEG found that global theta-gamma PAC was lower in patients with progressed MCI compared to MCI patients who remained stable, which was also correlated with Addenbrooke’s Cognitive Examination (ACE) scores [132]. Although more studies are needed to validate such findings, they do suggest that the pattern of oscillatory activity as measured by EEG and theta-gamma PAC analysis may potentially serve as a biomarker of disease progression [18, 132, 133].

## Altered neural activity dynamics in DS

DS results from the trisomy of chromosome 21 and is associated with a variety of cognitive comorbidities, such as intellectual disability and AD [134]. The vast majority of individuals with DS develop extracellular deposits of Aβ and intracellular accumulation of tau-containing NFTs by their mid-thirties to 40 years of age, which put this population at greater risk of early-onset AD [20, 135]. Indeed, the mean age of onset of clinical dementia in this population is about 55 years [136] and DS has been acknowledged as a genetic form of AD [24, 137, 138].

The molecular pathophysiology of DS-AD is not well defined. There is general acknowledgment that the extra copy of the gene encoding for amyloid precursor protein (*APP*), which is located on chromosome 21, is associated with increased susceptibility to AD [139-141]. Yet, recent preclinical findings have showed that the triplication of chromosome 21 genes other than *APP* may also increase Aβ aggregation, deposition of plaques, and worsen AD-related cognitive impairments [142].

Dementia has been found to be the cause of death for 70% of adults with DS [21], which is much higher than that in the general population. This continues to be the case even when considering recent work showing that, from 2004 to 2017, the percentage of older (> 67 years of age) adult decedents who received an all-cause dementia diagnosis increased substantially, from 34.7% in 2004 to 47.2% in 2017 [98, 143, 144]. Therefore, given the much younger age of dementia diagnosis in those with DS and the overwhelming percentage of those who will succumb to DS-AD in their 50’s and 60’s, there is a clear and urgent need for objective measures for the identification of prodromal stages of AD in persons with DS.

Current evidence shows that similar changes in AD-related biomarkers are found in individuals with DS-AD compared to those with sporadic and autosomal dominant forms of AD [24, 145, 146]. For instance, the likelihood of developing AD was up to 5 times higher for individuals with DS who had decreased Aβ_42_ plasma levels and Aβ_42_/Aβ_40_ ratio when compared to those with increased levels of such biomarkers [147]. Plasma levels of NfL was found to increase with age, as well as to inversely correlate with plasma Aβ_42/40_ ratio in a cohort of adults with DS [148]. Greater PET measurements of fibrillar Aβ were found in DS individuals with dementia than in those without dementia [149]. Importantly, it was recently reported that the natural history of AD-related biomarker changes in adults with DS was similar to that described for autosomal dominant AD [24], which suggests that DS-AD may share similar pathophysiology with AD in the general population [20].

Electrophysiological findings in persons with DS and AD are also consistent with those in individuals with AD without DS [150]. A cross-sectional study showed earlier age-related slowing of the alpha rhythm by up to a decade in DS subjects compared to cognitively healthy individuals [151]. Indeed, Murata and collaborators reported a significant age-related slowing of the mean frequency in individuals with DS (9.37 Hz in the 20's group *vs* 8.76 Hz in the 40's group), while this parameter only slightly decreased with age in the controls (9.74 Hz in the 20's group *vs* 9.53 Hz in the 60's group; no statistical difference) [152]. Given that alpha oscillation activity changes were also reported in prodromal stages of AD [108, 118, 153, 154], it has been hypothesized that these changes may indeed be considered as an early sign of AD-associated synaptic degeneration in adults with DS [151, 155, 156]. Individuals with DS and with no sign of cognitive decline or dementia showed increased power within the theta band, as suggestive of alpha slowing, when compared to subjects without DS [157-159]. Importantly, as disease progressed from MCI to AD in subjects with DS, the previous increased theta activity was replaced by an increase in the delta power [157]. This observation is particularly important since increased theta activity is widely accepted as an EEG marker of early-stage AD [108, 153, 154], whereas increased delta power seems to appear at later stages of the disease [108, 109, 160]. In addition, a less pronounced increase in alpha, beta, and gamma band activity in response to 12-Hz photic stimulation were found in subjects with DS without dementia compared to age-matched controls [159], similar to what has been typically found in patients with AD [120].

In sum, electrophysiological findings in persons with DS corroborate evidence from studies supporting the well-accepted notion in the AD research community that slowing of brain rhythm may be associated with the level of cognitive deterioration, and hence may serve as a biomarker to predict the onset of dementia [157, 161]. Yet, few studies in DS have focused on investigating cross-frequency interactions between different brain rhythms, and much of the existing evidence arises from preclinical findings [162-164].

## Electrophysiological AD-related phenotypes in animal models of AD and DS

Many AD-relevant phenotypes can be observed in mice overexpressing *App*, such as the Ts65Dn mouse model of DS [165]. The Ts65Dn mouse, which is the most widely studied mouse model of DS, is segmentally trisomic for approximately two thirds of the chromosome 21 orthologous region on mouse chromosome 16 [166]. This mouse model displays both triplication of genetic material and chromosomal aneuploidy, which are features seen in the large majority of individuals with DS. The trisomic region contains over 100 mouse orthologs to chromosome 21 genes, including the murine gene encoding for APP. Although Ts65Dn mice do not develop Aβ plaques and NFTs [167, 168], they do show increased levels of APP and its proteolytic products, such as Aβ_40_ and Aβ_42_ monomers and soluble Aβ oligomers [169-171]. Soluble oligomers of Aβ have been considered more toxic and pathogenic than plaques since they may affect synaptic structure and function at early stages of AD [172-174]. These oligomers have been found to inhibit LTP [175-178], enhance LTD, and reduce dendritic spine density in rodents [176]. Importantly, all of these synaptic changes have been described in Ts65Dn mice [179-183].

The Ts65Dn mouse also shows progressive, age-related degeneration of basal forebrain cholinergic neurons [167, 184]. This form of cholinergic neurodegeneration seems to contribute to poor performance of Ts65Dn mice in some hippocampal-dependent memory tasks [185-188] and correlates with increased *App* gene dosage and reduced retrograde axonal transport of nerve growth factor [184, 189, 190]. These findings are of interest because axonopathy and transport deficits have also been associated with AD pathogenesis [191, 192], and cholinergic neurons have been shown to undergo extensive degeneration even in mild cases of AD when compared to healthy control individuals [193-195].

Decline in cholinergic signaling may contribute to AD-related brain electrical activity alterations [72, 196], given that pharmacological enhancement of cholinergic transmission has been found to upregulate hippocampal theta activity in animal models of AD [123, 126, 197]. Indeed, impairments in theta oscillations have been consistently reported in several animal models of AD [123, 126, 197-199], and this phenomenon may anticipate performance deficits in hippocampal-dependent tasks [123, 199, 200]. Interestingly, Aβ deposition mostly occurs along neural networks with abnormal activity in both cognitively intact and AD subjects [201, 202], indicating that network abnormalities is an early event in AD pathogenesis [201].

There is growing evidence that the activity of interneurons, as well as the oscillatory network activities they regulate, are impaired in AD [110, 111, 203]. Findings from preclinical studies showed that Aβ-induced dysfunction of inhibitory interneurons may shift the activity of pyramidal neurons from normal to aberrantly synchronized [197, 204-206].

A decrease in rhythmic bursting activity of septo-hippocampal PV interneurons has been observed following hippocampal Aβ injections, which correlated with decreased theta power and impaired recognition memory performance in rats [207]. Overexpression of *App* and increased levels of Aβ peptides have also been found to impair gamma oscillations in both *in vitro* and *in vivo* preclinical studies [32, 126, 208, 209]. Such impairments have also been associated with dysfunctional PV basket cell signaling and learning/memory deficits in animal models of AD [110, 111, 210, 211]. This evidence is further supported by studies showing that enhancement of PV basket cell-dependent gamma activity restored neural and behavioral deficits in these animals [111, 211-214]. Additionally, hippocampal theta-gamma PAC has also been found to be impaired in preclinical models of AD (Table 1) [123-126, 128-131, 210, 212, 215-224], which may occur before or at an early stage of tau and Aβ pathologies [124, 125, 127, 128].

Inhibitory circuit dysfunction has been implicated as a contributing mechanism to DS pathophysiology [225]. Findings from preclinical studies suggest that an early excitation/inhibition imbalance leads to over-inhibition in neural circuits, which may contribute to changes in network activity of DS mouse models [162-164, 226, 227] (Table 1). For example, Dp1Tyb mice showed a general slowing of theta-band oscillations across the hippocampus and medial prefrontal cortex (mPFC), which was associated with slower decision-making in a spontaneous alternation task [162]. An increased hippocampal-mPFC theta coherence was also observed in these animals, which is in agreement with clinical findings showing increased synchrony between neural networks in persons with DS [228, 229].

**Table 1:** Cross-frequency phase-amplitude coupling alterations in animal models of AD and DS.

Reference	Animal model	Age (months)	Type of study	Brain area (s) recorded	Recording conditions	Brain oscillations studied	Type of alteration observed	Type of index used
Goutagny et al., 2013	TgCRND8 mice	1-month-old	*In vitro*	Hippocampus (subicular area)	Spontaneous LFP activity.	Theta (3-12 Hz) and fast gamma (120-250 Hz) oscillations.	Decrease in theta-fast gamma phase-amplitude coupling.	Tort et al., 2008
Ittner et al., 2014	APP23 mice	4-month-old	*In vivo*	Hippocampus	Spontaneous LFP activity was recorded during freely-roaming condition.	Theta (4-12 Hz) and gamma (25-100 Hz) oscillations.	Decrease in theta-gamma phase-amplitude coupling.	Tort et al., 2008
Kalweit et al., 2015	Aβ_(1-42)-_treated rats	6-month-old	*In vivo*	Hippocampus (dentate gyrus area)	LFP activity was recorded during high-frequency stimulation (HFS) of the perforant path-dentate gyrus synapse in freely-behaving rats.	Theta (4-10 Hz) and gamma (30-100 Hz) oscillations.	Decrease in theta-gamma phase-amplitude coupling.	Bruns and Eckhorn, 2004
Booth et al., 2016	rTg4510 mice	7- to 8-month-old	*In vivo*	Hippocampus (CA1 area)	LFP activity was recorded while mice runned from end to end in a linear track for appetitive rewards.	Theta (4-12 Hz) and gamma (25-120 Hz) oscillations.	Decrease in theta-gamma phase-amplitude coupling.	Canolty et al., 2006
Stoiljkovic et al., 2016	5xFAD mice	8-month-old	*In vivo*	Hippocampus (CA1 area)	Stimulation-induced (electrical stimulation of the nucleus pontis oralis) hippocampal LFP activity. Recordings were done under urethane anesthesia.	Theta (3-12 Hz), low (30-50 Hz) and high gamma (75-95 Hz) oscillations.	Decrease in theta-high gamma phase-amplitude coupling.	Tort et al., 2008
Zhang et al., 2016	APP knockout (APP-KO) mice	9-month-old	*In vivo*	Hippocampus (CA1 area), medial prefrontal cortex, and posterior parietal cortex	Spontaneous LFP activity was recorded during awake (freely-behaving) and REM sleep condition.	Theta (4-12 Hz), gamma (40-100 Hz) and fast gamma (120-160 Hz) oscillations.	Decreased parietal theta-gamma phase-amplitude coupling during awake and REM sleep. Decreased parietal theta-fast gamma phase-amplitude coupling during REM sleep. Decreased hippocampal theta-gamma phase-amplitude coupling during awake and REM sleep. Decreased prefrontal-hippocampal theta-gamma phase-amplitude coupling during awake.	Tort et al., 2008
Fontana et al., 2017	PS2APP mice	6- and 12-month-old	*In vivo*	Hippocampus (dentate gyrus area)	Spontaneous LFP activity was recorded under urethane anesthesia.	Theta (1.7-4.7 Hz), beta (10-25 Hz), slow gamma (25-40 Hz), fast gamma (45-90 Hz), and oscillations epsilon (110-190 Hz).	Increased theta-beta and theta-slow gamma phase-amplitude couplings (6-month-old). Decreased theta-epsilon phase-amplitude coupling (12-month-old).	Penny et al., 2008
Joo et al., 2017	TgF344-AD rats	9-month-old	*In vivo*	Somatosensory cortex	Spontaneous LFP activity was recorded during resting-state condition.	Theta (4-8 Hz) and gamma (30-140 Hz) oscillations.	Decrease in theta-gamma phase-amplitude coupling.	Canolty et al., 2006
Mably et al., 2017	3xTg mice	8- to 9-month-old	*In vivo*	Hippocampus (CA1 area)	LFP activity was recorded while mice unidirectionally runned around a circular track for appetitive rewards.	Theta (4-12 Hz), slow (25-40 Hz) and fast gamma (65-100 Hz) oscillations.	Decrease in theta-slow gamma phase-amplitude coupling.	Colgin et al., 2009
Nakazono et al., 2017	APP knock-in (APP-KI) mice	5-month-old	*In vivo*	Medial entorhinal cortex	Spontaneous LFP activity was recorded under urethane anesthesia.	Theta (5-10 Hz) and gamma (30-100 Hz) oscillations.	Decrease in theta-gamma phase-amplitude coupling.	Lengths of resultant vectors of theta phase distributions of gamma oscillation maxima were used as an index for theta-gamma coupling.
Tanninen et al., 2017	P301L-mutant human tau-expressing rats	not reported	*In vivo*	Hippocampus and medial prefrontal cortex	LFP activity was recorded while rats performed trace eyeblink conditioning (TEBC).	Theta (6-12 Hz) and gamma (30-100 Hz) oscillations.	Increased prefrontal theta-gamma phase-amplitude coupling. Decreased prefrontal-hippocampal theta-gamma phase-amplitude coupling.	Tort et al., 2008
Bazzigaluppi et al., 2018	TgF344-AD rats	9-month-old	*In vivo*	Hippocampus and medial prefrontal cortex	Spontaneous LFP activity was recorded during resting-state condition.	Theta (3-9 Hz), low (30-58 Hz) and high gamma (62-120 Hz) oscillations.	Decrease in theta-high gamma phase-amplitude coupling in both hippocampus and medial prefrontal cortex.	Canolty et al., 2006
Mondragón-Rodríguez et al., 2018	J20 mice	1-month-old	*In vitro*	Hippocampus (subicular area)	Spontaneous LFP activity.	Theta (2-12 Hz), slow (25-55 Hz) and fast gamma (150-250 Hz) oscillations.	Decrease in both theta-slow and theta-fast gamma phase-amplitude couplings.	Tort et al., 2008
Etter et al., 2019	J20 mice	6-month-old	*In vivo*	Hippocampus (CA1 area)	LFP activity was recorded while mice freely explored a circular platform.	Theta (6-12 Hz) and gamma (30-60 Hz) oscillations.	Decrease in hippocampal theta-slow gamma phase-amplitude coupling.	Belluscio et al., 2012 and Tort et al., 2008
Leparulo et al., 2019	PS2APP, PS2.30H, and APPSwe mice	3- and 6-month-old	*In vivo*	Hippocampus (CA1 area) and posterior parietal cortex	Spontaneous LFP activity was recorded under a mixture of urethane and xylazine/tiletamine-zolazepam anesthesia.	Slow (SO; 0.1-1.7 Hz) and fast gamma (45-90 Hz) oscillations.	Decreased parietal-hippocampal SO-fast gamma coupling in 3- and 6-month-old PS2.30H mice. Decreased parietal-hippocampal SO-fast gamma coupling in 6-month-old PS2APP and APPSwe mice.	Penny et al., 2008
Stoiljkovic et al., 2019	TgF344-AD rats	12-month-old	*In vivo*	Hippocampus (CA1 area)	Stimulation-induced (electrical stimulation of the nucleus pontis oralis) hippocampal LFP activity. Recordings were done under urethane anesthesia.	Theta (3-9 Hz), low (30-55 Hz), and high gamma (65-95 Hz) oscillations.	Decrease in both theta-low and -high gamma phase-amplitude coupling.	Tort et al., 2008
Ahnaou et al., 2020	P301S mice	3-, 6-, and 9-month-old	*In vivo*	Hippocampus (CA1 area), olfactory bulb, frontal cortex, and lateral entorhinal cortex	Spontaneous LFP activity was recorded in freely-behaving mice.	Theta (4-8 Hz) and gamma (40-100 Hz) oscillations.	Decreased theta-gamma phase-amplitude coupling in the olfactory bulb.	Canolty et al., 2006
Alemany-González et al., 2020	Ts65Dn mice	2- to 3-month-old	*In vivo*	Hippocampus (CA1 area) and medial prefrontal cortex	Spontaneous LFP activity was recorded during quiet wakefulness condition.	Delta (3-5 Hz), theta (6-12 Hz), high gamma (80-120 Hz), and high frequency (100-200 Hz) oscillations.	Increased prefrontal delta-high gamma phase-amplitude coupling and increased hippocampal theta-high frequency phase-amplitude coupling.	Tort et al., 2008
Chang et al., 2020	Dp1Tyb and Dp10Yey mice	3- to 9-month-old	*In vivo*	Hippocampus and medial prefrontal cortex	LFP activity was recorded while mice performed a spontaneous alternation task.	Theta (6-12 Hz), low (60-120 Hz), and high gamma (140-160 Hz) oscillations.	Increased hippocampal theta-high gamma phase-amplitude coupling and increased theta-band coherence between the hippocampus and mPFC in Dp1Tyb mice. Decreased hippocampal theta-low gamma phase-amplitude coupling in Dp10Yey mice.	Colgin et al., 2009
Kumari et al., 2020	APP/PS1 mice	6-month-old	*In vivo*	Hippocampus (dentate gyrus area) and dentate gyrus-perforant pathway	LFP activity was recorded following electrical stimulation of the perforant pathway. Recordings were done under urethane anesthesia.	Theta (3-8 Hz), low (30-50 Hz), and high gamma (50-100 Hz) oscillations.	Decreased theta-low and -high gamma phase-amplitude coupling in both dentate gyrus area and dentate gyrus-perforant pathway.	Cheng et al., 2016
Park et al., 2021	5xFAD mice	6-month-old	*In vivo*	Frontal and parietal areas	Spontaneous EEG was recorded in freely-behaving mice.	Delta (2-5 Hz), theta (5-8 Hz) and gamma (30-80 Hz) oscillations.	Decreased frontal-parietal delta-gamma phase-amplitude coupling. Decreased parietal-frontal theta-gamma phase-amplitude coupling.	Combrisson et al., 2020
Leparulo et al., 2022	PS2APP mice	6- and 12-month-old	*In vivo*	Hippocampus (CA1 area) and posterior parietal cortex	Spontaneous LFP activity was recorded under a mixture of urethane and xylazine/tiletamine-zolazepam anesthesia.	Slow (SO; 0.1-1.7 Hz) and fast gamma (45-90 Hz) oscillations.	Decreased hippocampal-parietal SO-fast gamma coupling. Decreased parietal-hippocampal SO-fast gamma coupling.	Penny et al., 2008
Tok et al., 2022	Tau-seeded APP-KI_NL-G-F_ and APP-KI^NL^ mice	3- and 6-month-old	*In vivo*	Hippocampus (CA1 area)	Spontaneous LFP activity was recorded in freely-behaving mice.	Theta-1 (4-6 Hz), theta-2 (6-8 Hz), low gamma (30-50 Hz) and high gamma (51-80 Hz) oscillations.	Decreased theta2-high gamma phase-amplitude coupling in both mouse models and age groups one day after injection.	Tort et al., 2008

Mouse models of DS also showed altered hippocampal theta-phase modulation of low and high gamma amplitude during rest and behavioral performance [162, 163]. Recently, it has been reported that PV basket cells lost their classical fast-spiking phenotype and showed increased excitability in mPFC slices from Ts65Dn mice [164]. Conversely, an enhancement in the dendritic inhibitory loop involving somatostatin-positive (SST) Martinotti cells and pyramidal neurons was also observed in mPFC slices from these animals. Such alterations likely contributed to reduce spiking activity in pyramidal neurons, which were also more strongly phase-locked to fast oscillations [164].

Although preclinical studies have shed some light on the potential links between PAC alterations and brain phenotypes associated with cognitive deficits in DS, the underlying mechanisms leading to these pathophysiological processes remain to be further elucidated.

## Looking forward: strengths and limitations of the potential utility of cross-frequency phase-amplitude coupling analysis as an adjuvant diagnostic tool in DS-AD

Despite of growing preclinical and clinical evidence supporting the use of EEG to probe the effects of AD pathology on neurophysiological parameters, currently applied diagnostic criteria do not yet support the application of EEG-based biomarkers in AD clinical practice [230-232]. A similar scenario is observed in the research field aiming to identify biomarkers for AD in DS. For example, the protocol of the most ambitious AD biomarker initiative to date in adults with DS, the Alzheimer’s Biomarker Consortium-Down Syndrome (ABC-DS), includes advanced MRI- but not EEG-derived measures as outcome measures of functional connectivity [233].

Even with the limitations of preclinical experimentation (as discussed below), animal models have been employed in the study of the association between AD pathology and its neurophysiological effects (as measured by EEG) on brain oscillations and network dynamics [110]. Importantly, a robust body of preclinical evidence on AD-associated electrophysiological alterations parallel EEG data recording from AD patients. Similarly, previous findings of impaired PAC in mouse models of DS mirror those observed in both animal models and subjects with AD [121-125, 127, 128]. Furthermore, an association has been found between altered theta-gamma PAC and higher incidence of cognitive decline that later converts to AD and dementia [18, 19, 133]. Therefore, we propose that future research efforts should be undertaken toward the study of network-based biomarkers, such as CFC/PAC, as an adjuvant diagnostic tool in DS-AD. In particular, we speculate that changes in network interactions as assessed by CFC analysis may have a role on identifying individuals with DS at a very early stage of AD. In addition, we have good reason to suspect that this field of research could potentially give important clues to the biophysical properties and pathophysiological mechanisms underlying the progressive neurodegenerative processes in DS-AD.

Electrophysiological technology has great appeal as a biomarker for several reasons. This technique is generally non-invasive, low-cost, widely available, and ease to deploy (including training requirements for staff) in both clinical practice and clinical trials. In contrast, other approaches, such as CSF and neuroimaging biomarkers, demand higher technical expertise and have operational limitations (i.e., they are not broadly available), besides being considered “more than minimal risk” by regulatory agencies (e.g., lumbar puncture for obtaining CSF or the use of radiolabeled ligands for PET scan). Additionally, EEG recordings may also be better tolerated by persons with intellectual disability than other investigative modalities [23, 234].

Compared to other technologies that also measure brain function, such as functional MRI (fMRI), EEG is less sensitive to motion, and it is far less expensive. In addition, while fMRI records blood oxygen level-dependent (BOLD) responses, and thus provides an indirect measure of neuronal activity, EEG captures neuronal activity at its natural high-temporal resolution, and thus may provide better accuracy than fMRI in detecting early changes in brain dynamics associated with AD-related cognitive decline.

EEG-based biomarkers may also have the potential to aid in advancing the development, clinical implementation, and monitoring of novel drug candidates against AD. Recently, a double-blind, placebo-controlled trial used EEG-derived measures to evaluate the safety, tolerability, and efficacy of the glutaminyl cyclase inhibitor PQ912 in patients with MCI or mild dementia due to AD [235]. According to this study, significant PQ912-induced changes in theta power indicate that EEG may be considered a suitable biomarker for proof-of-concept studies [235].

The utility of EEG recordings as a potential longitudinal measure of treatment efficacy has been explored for decades. For example, EEG spectral analysis showed a power decrease in delta [236] and theta [236, 237] frequency bands during rivastigmine treatment, which was also associated with mini-mental state score improvements. In addition, a decrease in delta amplitude, along with an increase in alpha and beta activity, was observed following treatment with donepezil, which is a clinically approved cholinesterase inhibitor for AD treatment [238]. At the preclinical level, the acute administration of donepezil has been found to modulate the strength of theta-gamma PAC [197]. Together, these findings support the use of network-level biomarkers as a potential approach for predicting treatment outcomes, as well as for assessing effectiveness of therapeutic strategies with disease-modifying potential in AD.

There are many limitations that still need to be addressed before considering the general application of EEG-based biomarkers such as PAC as an adjuvant tool for detecting DS-AD. A partial list of the necessary steps to achieve this goal is provided in the following paragraphs.

First, further biological validation is needed by larger studies, as well as by studies involving different animal models, given that there is currently no animal model that faithfully reproduces the whole phenotypic spectrum of DS. In addition, how well animal models of DS can mirror PAC alterations in the DS clinical setting also remains to be determined.

Second, correlations of EEG findings with well-established AD biomarkers (such as CSF, plasma and neuroimaging biomarkers), as well as with markers of cognitive function, should be considered in future preclinical and clinical studies. The longitudinal investigation of changes in EEG-based biomarkers and cognitive outcome measures may provide key information on time sequence of transition from cognitive health to cognitive impairment and decline.

Third, standardization of operating procedures for EEG recording and data analysis will be needed to enable valid comparisons between basic and human research laboratories, as well as with clinical centers. For example, changes in PAC strength depend on cognitive demands [37] and performance [6]. As illustrated in Table 1, many methods for PAC estimation have been reported in the literature [8, 37, 42, 60, 61, 239-241], although some metrics seem more suitable for assessing the existence of the coupling as well as its magnitude [43, 241].

Fourth, because of the phenotypic variability in DS-related cognitive traits and the presence of potential confounding factors (such as DS-related comorbidities), it is likely that a combination of biomarker modalities will turn out to be a more effective way to discriminate between DS and DS-AD. While pathophysiological markers (such as amyloid PET and CSF Aβ_42_, NfL, and phosphorylated-tau) have been suggested to directly provide *in vivo* evidence of AD pathology, topographical markers (such as hippocampal atrophy assessed by MRI and cortical hypometabolism measured by FDG PET) characterize downstream brain changes generally induced by AD pathology [242]. EEG-based markers may thus serve as part of the topographical evidence by detecting AD-related synaptic and neural dysfunction. In addition, studies involving multiple biomarker modalities may also benefit from the use of EEG-based biomarkers as a means to improve the predictive power of other well-known AD biomarkers [243-245].

## Conclusions

In this Perspective, we have provided the rationale for pursuing PAC as a potential adjuvant tool in the study of the pathophysiological processes underlying brain dynamics in DS-AD, as well as in the identification of EEG-based biomarkers with potential diagnostic and prognostic utilities in DS-AD. From the perspective of basic science, the continued investigation of neural network dynamics in mouse models of DS may aid in enhancing our understanding of how AD pathology evolves in persons with DS, as well as in extending knowledge of early neurophysiological signatures of DS-AD that could potentially be of clinical use. From the perspective of clinical practice, developing non-invasive, low-cost, and easily obtainable biomarkers (such as those based on EEG) for the diagnosis of AD may be an important step toward the identification of individuals at the preclinical or prodromal stage of the disease, especially in the most susceptible populations as the case of DS.

Identifying individuals with DS at the very earliest stages of AD is vital for maximizing the efficacy of therapeutic interventions to mitigate cognitive decline due to AD. In addition, early identification of individuals with DS at risk of developing AD would allow them to be recruited in intervention trials aimed at testing disease-modifying drugs whose therapeutic goals are to prevent or slow AD-related cognitive decline. EEG-based markers might also prove to be useful in clinical routine practice to monitor therapy efficacy not only in people with DS-AD, but also in those with AD in the general population. Yet, it remains to be determined how EEG-based biomarkers are associated with other biomarkers of DS-AD, as well as with the onset of AD-related signs and symptoms. Although much work remains to be done, we encourage a continuous debate on the potential utility of PAC measures, not only as an EEG-based biomarker or surrogate marker, but also as a tool for studying the pathophysiological processes underlying DS-AD.
